# Unique and shared transcriptomic signatures underlying localized scleroderma pathogenesis identified using interpretable machine learning

**DOI:** 10.1172/jci.insight.185758

**Published:** 2025-04-08

**Authors:** Aaron BI Rosen, Anwesha Sanyal, Theresa Hutchins, Giffin Werner, Jacob S. Berkowitz, Tracy Tabib, Robert Lafyatis, Heidi Jacobe, Jishnu Das, Kathryn S. Torok

**Affiliations:** 1 Center for Systems Immunology, Departments of Immunology and Computational & Systems Biology;; 2 Department of Pediatrics; and; 3 Division of Rheumatology and Clinical Immunology, Department of Medicine, University of Pittsburgh, Pittsburgh, Pennsylvania, USA.; 4 Department of Dermatology, University of Texas Southwestern, Dallas, Texas, USA.

**Keywords:** Autoimmunity, Immunology, Autoimmune diseases, Bioinformatics

## Abstract

Using transcriptomic profiling at single-cell resolution, we investigated cell-intrinsic and cell-extrinsic signatures associated with pathogenesis and inflammation-driven fibrosis in both adult and pediatric patients with localized scleroderma (LS). We performed single-cell RNA-Seq on adult and pediatric patients with LS and healthy controls. We then analyzed the single-cell RNA-Seq data using an interpretable factor analysis machine learning framework, significant latent factor interaction discovery and exploration (SLIDE), which moves beyond predictive biomarkers to infer latent factors underlying LS pathophysiology. SLIDE is a recently developed latent factor regression-based framework that comes with rigorous statistical guarantees regarding identifiability of the latent factors, corresponding inference, and FDR control. We found distinct differences in the characteristics and complexity in the molecular signatures between adult and pediatric LS. SLIDE identified cell type–specific determinants of LS associated with age and severity and revealed insights into signaling mechanisms shared between LS and systemic sclerosis (SSc), as well as differences in onset of the disease in the pediatric compared with adult population. Our analyses recapitulate known drivers of LS pathology and identify cellular signaling modules that stratify LS subtypes and define a shared signaling axis with SSc.

## Abstract 2

Using machine learning to elucidate the cellular and molecular bases of disease pathogenesis in localized scleroderma in adult and pediatric populations

## Graphical abstract

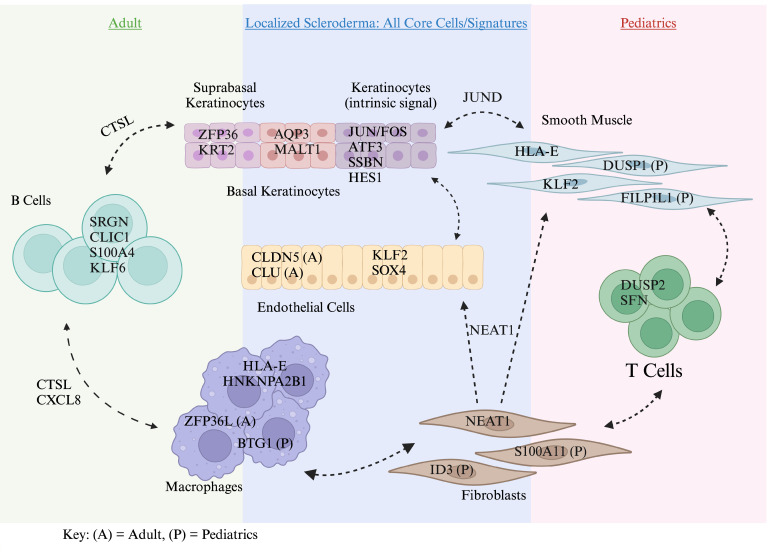

## Introduction

Morphea, also referred to as localized scleroderma (LS), is a complex autoimmune disease and presents clinically with a combination of inflammation and fibrosis involving the skin and deeper tissues, affecting muscle to the central nervous system and causing significant disability, which is often more severe in children (1–4). LS is often referred to as a systemic autoimmune disorder since there is a prevalent family history of autoimmune disease (3), and patients studied have shared HLA types with rheumatoid arthritis (5); high frequency of autoantibodies (6, 7); and elevated circulating chemokines and cytokines associated with Th cell, IFNG, and other inflammatory pathways (8–11). Studies in skin and peripheral blood of patients with LS via microarray, RNA-Seq, and tissue staining (12–14) have supported an IFN-associated inflammatory phenotype. Globally LS is an understudied disease, with much of its pathophysiology extrapolated from systemic sclerosis (SSc) since the histopathologic findings are nearly identical (15). Although the key players in the pathogenesis of LS remain elusive, close inspection of inflammatory lymphocytic and macrophage infiltrate with collagen and fibroblast deposition supports the notion that LS is a disease of inflammation-driven fibrosis (16).

More recently, single-cell RNA-Seq (scRNA-Seq) has provided further clues to the drivers of LS by deconvoluting the signatures identified in bulk RNA sequencing. In prior work, we have elucidated key cellular programs involving fibroblasts that are elevated in LS compared with healthy controls (i.e., *CCL19*/*APOE*, *CXCL2*/*IRF1*, *CXADR*/*GATA3*). These programs may be responsible for driving the disease, with ligand-receptor analyses supporting a strong communication with macrophages to promote a myofibroblast-like phenotype (*SFRP4*/*PRSS23*) (17). The interactions between macrophages and fibroblasts observed in LS parallel those found in SSc, including some common subsets and signatures, though LS also involves several distinct cell subsets (18, 19). These are the initial findings describing LS pathogenesis; further investigation is needed in LS research to explore the heterogeneous cell types identified in scRNA-Seq, such as endothelial cells, epithelial cells, T cells, keratinocytes, and eccrine cells. Specifically, unbiased identification of cell-intrinsic and cell-extrinsic interactions involving immune and nonimmune cells is needed.

Recent work by our group, using predictive and interpretable machine learning approaches, uncovered some players in SSc pathogenesis from similar heterogenous cell datasets of the skin (20, 21). Specifically, a keratinocyte-centric signature and a novel mechanism involving altered HLA signaling in myeloid cells were identified in adult patients with SSc (20). In this current study, we sought to use LS skin-derived scRNA-Seq coupled with a recently developed interpretable latent factor regression approach, significant latent factor interaction discovery and exploration (SLIDE), to delve deeper into cell type–specific changes that characterize patients with LS across the disease spectrum regarding age of onset and disease severity.

## Results

### Transcriptomic profiling of patients with LS and healthy individuals.

**Figure 1:**
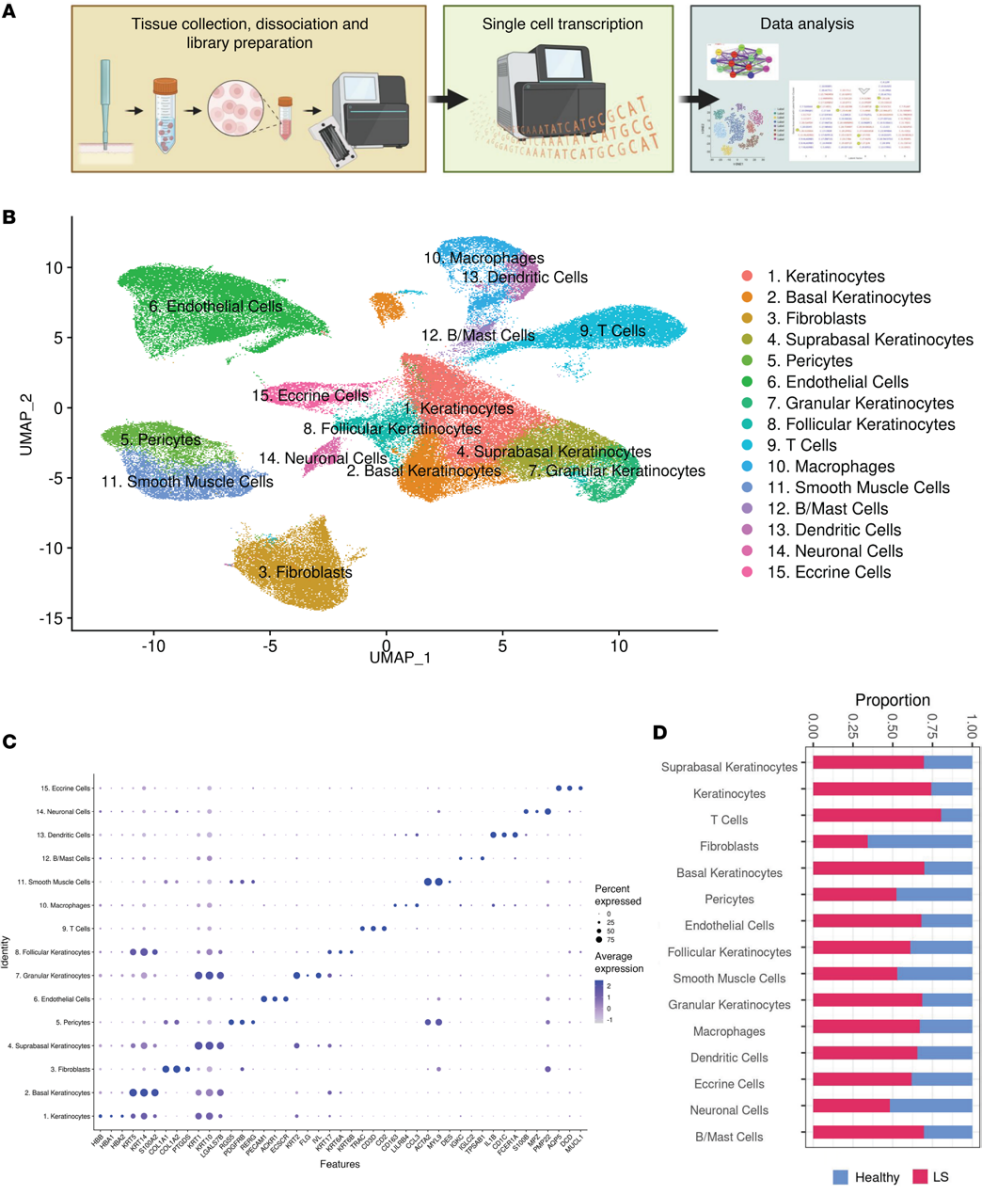
scRNA-Seq defines cell populations in the skin of patients with LS compared with age- and sex-matched healthy controls. (**A**) The 3 major steps of the pipeline are illustrated: tissue collection, dissociation, and library preparation; single-cell transcription; and data analysis. (**B**) The uniform manifold approximation and projection (UMAP) of the 122,809 cells derived from the skin of 27 LS samples and 17 healthy samples is displayed, with the final clustering into 15 cell type subclusters. (**C**) The marker genes specific to each cell cluster that led to the final cellular annotations are displayed. (**D**) The proportions of LS and healthy that comprise each of the 15 subclusters are illustrated, with overall higher proportions of inflammatory cells (i.e., T cells and B cells) and stromal cells (keratinocytes) in LS.

We analyzed single-cell transcriptomic profiles from 27 patients with LS (14 pediatric, 13 adult) and 17 healthy controls (9 pediatric, 8 adult) (Supplemental Table 1; supplemental material available online with this article; https://doi.org/10.1172/jci.insight.185758DS1). Unsupervised clustering with Seurat produced 32 cell clusters (Supplemental Figure 1), all of which included cells from both LS as well as healthy samples. These were then annotated to different cell types based on marker genes, and these incorporated most of the immune and nonimmune cell populations of interest, including lymphocyte, fibroblast, and keratinocyte subpopulations (17). Analyses were performed using these clusters, but for easier visualization we condensed these clusters into 15 main cell types (Figure 1B). When we compared expression of lineage marker genes, we saw our clusters had good correspondence to specific cell types, with little overlap between clusters, with appropriate gene annotations per cluster (Figure 1C). A comparison of the proportion of cell populations among LS compared with healthy controls suggests several immune and nonimmune/stromal cell types were elevated in LS, such as T cells and macrophages and several types of keratinocytes, respectively (Figure 1D).

### Identifying cell-specific transcriptomic signatures underlying the pathogenesis of LS in adult and pediatric individuals.

**Figure 2:**
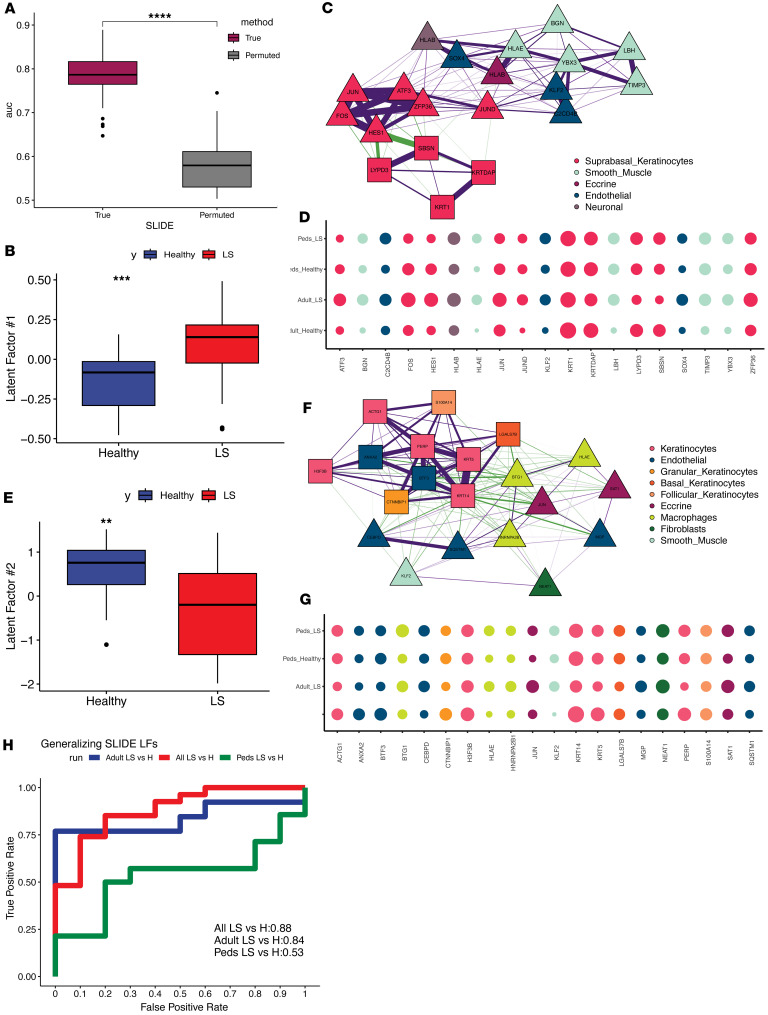
Cell-specific expression modules differentiate patients with LS from healthy controls. (**A**) SLIDE model performance measured by area under the receiver operating characteristics curve (AUC) using k-fold cross-validation. Significance assessed using permutation testing (negative control). Asterisks indicate Wilcoxon *P* < 0.0001. (**B**) Levels of significant latent factor 1 in LS and healthy patients. Asterisks indicate Wilcoxon *P* < 0.001. (**C**) Correlation network representation of significant latent factor 1. Purple and green edges indicate positive and negative correlations, respectively. Triangles indicate genes with higher expression in LS, and squares indicate genes with higher expression in control. (**D**) Bubble plots showing expression of genes in significant latent factor 1. Bubble size indicates average expression for within each group. (**E**) Levels of significant latent factor 2 in LS and healthy patients. Asterisks indicate Wilcoxon *P* < 0.01. (**F**) Correlation network representation of significant latent factor 2. (**G**) Bubble plots showing expression of genes in significant latent factor 2. (**H**) AUCs for classifying each group from healthy controls. Box plots show the interquartile range, median (line), and minimum and maximum (whiskers).

We reasoned that the heterogeneity and complexity of LS require a complex multivariate approach to identify transcriptomic signatures that distinguish between patients with LS and controls. While most machine learning approaches for high dimensional multiomics data identify only predictive biomarkers, we recently developed a latent factor regression approach, SLIDE, that comes with rigorous statistical properties and provides inference of actual pathophysiological processes beyond the identification of correlates alone. The SLIDE model, built on scRNA-Seq data from these 44 individuals (27 LS and 17 controls), provided discrimination between patients with LS and controls in a rigorous k-fold cross-validation framework with permutation testing (Figure 2A and Supplemental Table 2). The model identified 4 latent factors (context-specific coexpression modules), of which we further investigated the 2 most significant latent factors, 1 composed of a more compact cell-intrinsic module (Figure 2, B–D) and 1 multicellular, cell-extrinsic module (Figure 2, E–G). For the cell-intrinsic latent factor, we saw highly correlated upregulation of inflammatory markers (i.e., *HLAE*) in smooth muscle, as well as a separate cluster of proapoptotic genes (*JUND*, *FOS*, *HES1*, *ZFP36*) in suprabasal keratinocytes (Figure 2C). Interestingly, the keratinocyte-centric communication and upregulation of genes in keratinocytes (suprabasal dominant in LS, while slightly more basal dominant in SSc) strongly recapitulate recently published findings in SSc (21, 22). The cell-extrinsic latent factor similarly separated patients with LS and controls (Figure 2E) but included correlated gene expression between keratinocytes, macrophages, endothelial cells, and fibroblasts (Figure 2F). Patients with LS had enhanced *HLAE* in macrophages, *CEBPD* in endothelial cells, and upregulated *NEAT1* in fibroblasts. NEAT1 is known to play roles in promoting fibrosis through TGF-β–dependent pathways, cellular damage, and cell proliferation. As expected, our analysis highlights a central role for fibroblasts in overall disease progression (17). However, interestingly, most genes in the significant latent factors reflected relationships in either the adult or the pediatric population but not both (Figure 2G). Only a handful of genes were consistently altered in a similar fashion in both adult and pediatric populations, suggesting key differences between these 2 groups.

To better dissect this heterogeneity, we applied the learned latent factors to separately discriminate either adult or pediatric patients with LS from corresponding controls. While the model performed very well for the adult subset, it had poor performance for the pediatric subset (Figure 2H).

### Transcriptomic signatures specific to adult-onset LS.

**Figure 3:**
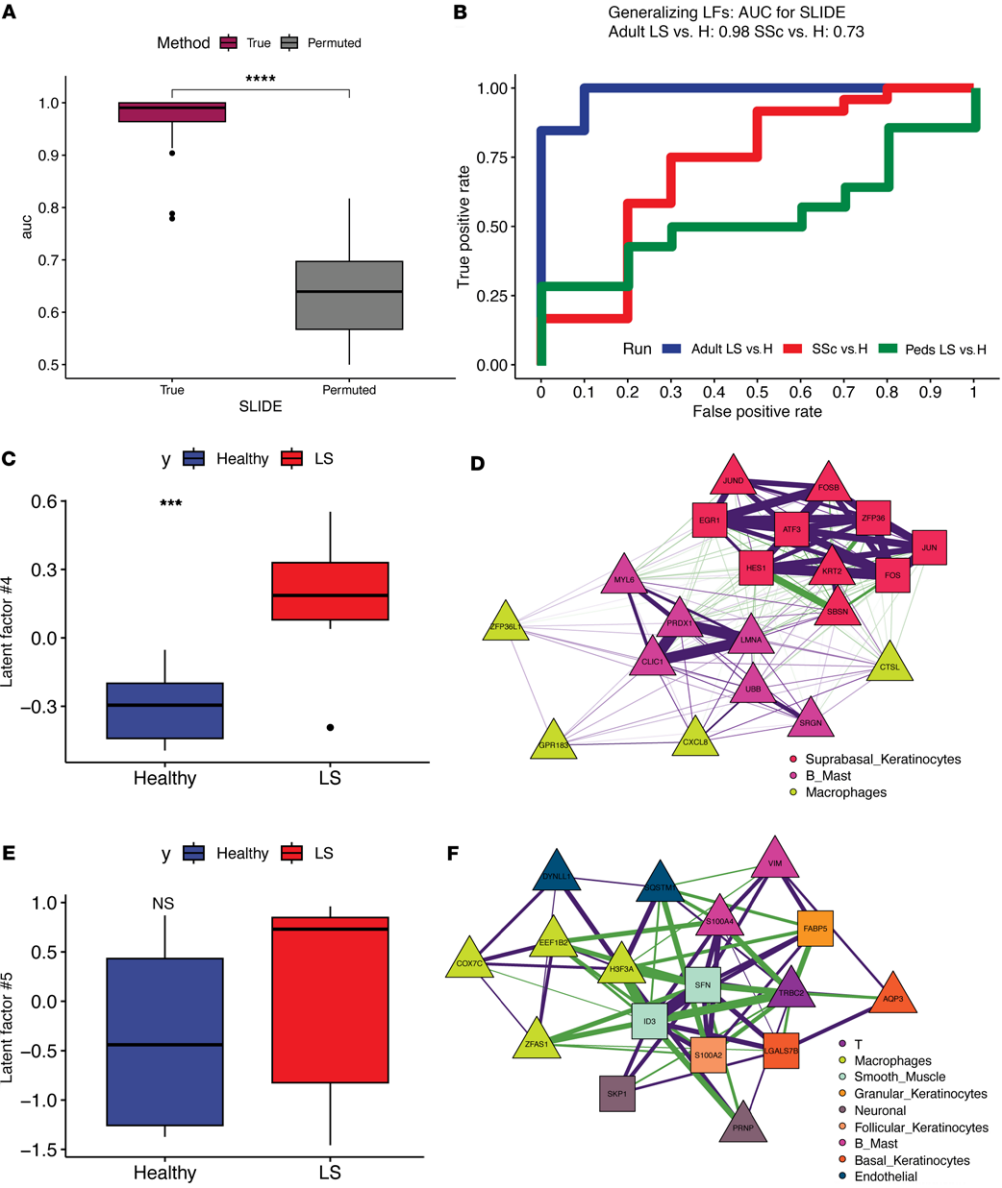
Cell-specific expression modules in adult LS resemble expression in SSc. (**A**) SLIDE model performance measured by AUC using k-fold cross-validation. Significance assessed using permutation testing (negative control). Asterisks indicate Wilcoxon *P* < 0.0001. (**B**) Latent factors trained to classify adult patients with LS can classify patients with SSc in cross-prediction. Cross-prediction AUCs for classifying each group from healthy controls. (**C**) Levels of significant latent factor 4 in adult patients with LS and healthy controls. Asterisks indicate Wilcoxon *P* < 0.001. (**D**) Correlation network representation of significant latent factor 4. Purple and green edges indicate positive and negative correlations, respectively. Triangles indicate genes with higher expression in LS, and squares indicate genes with higher expression in control. (**E**) Levels of significant latent factor 5 in adult patients with LS and healthy controls. (**F**) Correlation network representation of significant latent factor 5. Box plots show the interquartile range, median (line), and minimum and maximum (whiskers).

This disparity between adult and pediatric performance led us to delve further into the generalizability of these signatures. Given the heterogeneity across adult and pediatric populations, we reasoned that models specific to these 2 populations would provide more nuanced insights into the basis of pathogenesis**.** Unsurprisingly, our adult-specific SLIDE model provided excellent discrimination between patients with LS and controls and had over approximately 10% improvement in performance compared with our combined model (Figure 3A). We also tested whether this model could classify adults with SSc and controls whom we have previously described. Surprisingly, this model provided good discrimination for patients with SSc and controls (Figure 3B). Together, these results suggest that the transcriptomic signatures that capture primarily adult LS versus control differences generalize to the differences between adult SSc and control better than they do to pediatric LS and control. Also unsurprisingly, these latent factors were specific to adults and provided minimal discrimination for the pediatric population (Figure 3B). SLIDE found 5 significant latent factors, of which we focused on the 2 most significant ones. In these latent factors, we saw high discriminative power in the gene expression of suprabasal keratinocytes, B/mast cells, and macrophages (Figure 3, C and D, and Supplemental Table 3). Macrophages in LS showed higher expression of cycling- and adhesion-related genes, including *CTSL*, *CXCL8*, and *ZFP36L1*. B cells and mast cells also showed involvement in the cell-extrinsic network with an upregulation in *SRGN* and *CLIC1* in LS. The keratinocyte module in adults closely resembled the cell-intrinsic keratinocyte module in the combined model (adult and pediatric LS; *JUND*, *FOS*, *HES1*, *ZFP36*), which is striking considering that adult and pediatric patients are roughly matched in cohort size, so the retention of this module is not due simply to sample size (Figure 3D). There was an upregulation of *KRT2* and *AQP3* in the suprabasal and basal keratinocytes, respectively, in the adult patients with LS. We also highlighted a multicellular latent factor that consists of coordinated expression of alarmins, *S100A4* in B/mast cells and *S100A2* in granular keratinocytes, that correlated with decreased *ID3* and *SFN* in LS smooth muscle cells, which in turn associated with increased *ZFAS1* and *H3F3A* in macrophages (Figure 3, E and F). While most targets were associated with increased expression in adult LS samples, we also saw a few markers with decreased expression in LS. Notably, *FABP5* in granular keratinocytes and *LGALS7B* in basal keratinocytes were both decreased in adult patients with LS, suggesting additional physical, metabolic, and immunologic disruptions that may promote disease.

### Differences in transcriptomic signatures between adult and pediatric LS.

**Figure 4:**
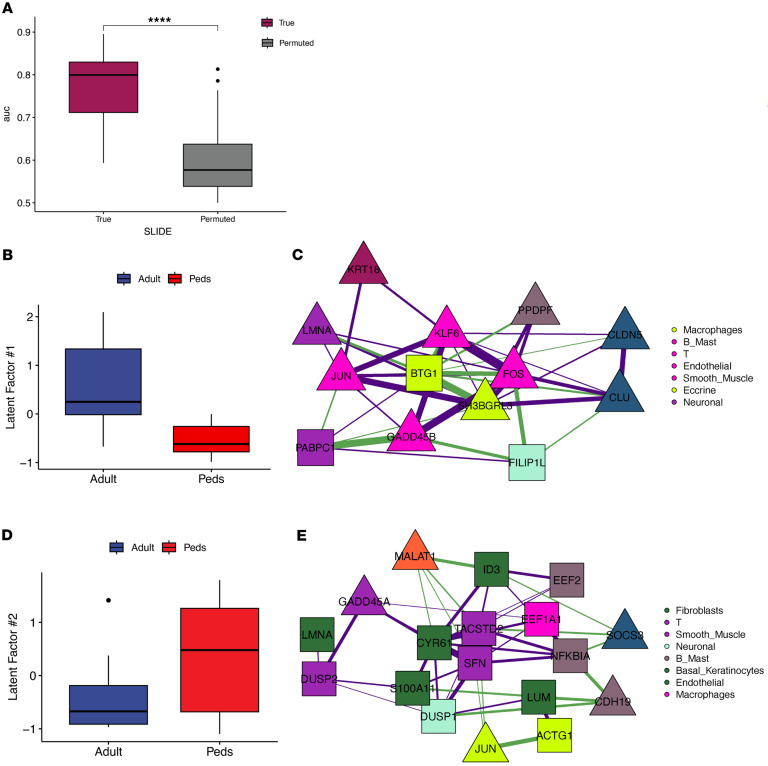
Adult and pediatric transcriptomic signatures in LS. (**A**) SLIDE model performance measured by AUC using k-fold cross-validation. Significance assessed using permutation testing (negative control). Asterisks indicate Wilcoxon *P* < 0.0001. (**B**) Levels of significant latent factor 1 in adult LS and pediatric LS. (**C**) Correlation network representation of significant latent factor 1. Purple and green edges indicate positive and negative correlations, respectively. Triangles indicate genes with higher expression in adult LS, and squares indicate genes with higher expression in pediatric LS. (**D**) Levels of significant latent factor 2 in adult LS and pediatric LS. (**E**) Correlation network representation of significant latent factor 2. Box plots show the interquartile range, median (line), and minimum and maximum (whiskers).

There were key differences between the combined and adult-only models, further verifying that pediatric LS is very different from adult LS. The primary common aspect was a keratinocyte-centric module, but our models indicate most other signatures are different between adult and pediatric populations. This agrees well with previous work by others and our group showing that LS subtype and outcome are distinctly different between adult and pediatric onset (2, 23, 24). To better characterize these differences between adult and pediatric LS, we used SLIDE to discriminate age-specific LS (Figure 4A). SLIDE identified 6 significant latent factors (Supplemental Table 4), of which we focused on the 2 most significant ones. In 1 significant latent factor, we noticed highly correlated gene expression between B/mast cells, macrophages, and endothelial cells (Figure 4, B and C). This latent factor suggests that upregulated *KRT18*, and *FOS* and *JUN* between eccrine and B/mast cells, respectively, are unique to adult LS, along with upregulated *MALAT1* in basal keratinocytes. We also highlighted a latent factor that was particularly informative of gene expression in pediatric LS (Figure 4D). Within this latent factor, there were high correlations between gene expression in T cells, fibroblasts, and smooth muscle cells (Figure 4E). There were particularly strong correlations between *FLIP1* and *DUSP1* upregulated in smooth muscle cells, *TACSTD2* in T cells, and *S100A11* in fibroblasts. Overall, LS in pediatric onset had fewer fibrotic genes and more inflammatory genes (upregulated *SFN* and *ID3* in T cells and fibroblasts). The main cellular circuits in pediatric LS are T cells, fibroblasts, and smooth muscle cells compared with the adult LS model, which demonstrates more upregulated pro-fibrotic factors among keratinocytes, macrophages, and B/mast cells as the key cellular players.

To account for the potential influence of anatomical site variability on molecular signatures, we performed a grouped cross-validation, where we used 1 anatomical site for model training and then evaluated the model’s performance for a different site holdout/test set. This allowed us to examine the robustness of our model across different body regions and mitigate potential confounding effects due to anatomical differences. We grouped body locations into 2 general categories, extremity (upper/lower) and trunk, for this holdout/test set analysis. Our results indicated strong performance (test AUCs = 0.74 and 0.91 for trunk and extremities, respectively) in discriminating between adult and pediatric LS when trained on samples from 1 anatomical site and tested on a completely different anatomical site. This cross-prediction analysis (across held-out anatomical sites) verifies that the observed differences between pediatric and adult LS are likely driven by age-related mechanisms and not by anatomical factors.

### Cell-intrinsic and cell-extrinsic models of LS severity.

**Figure 5:**
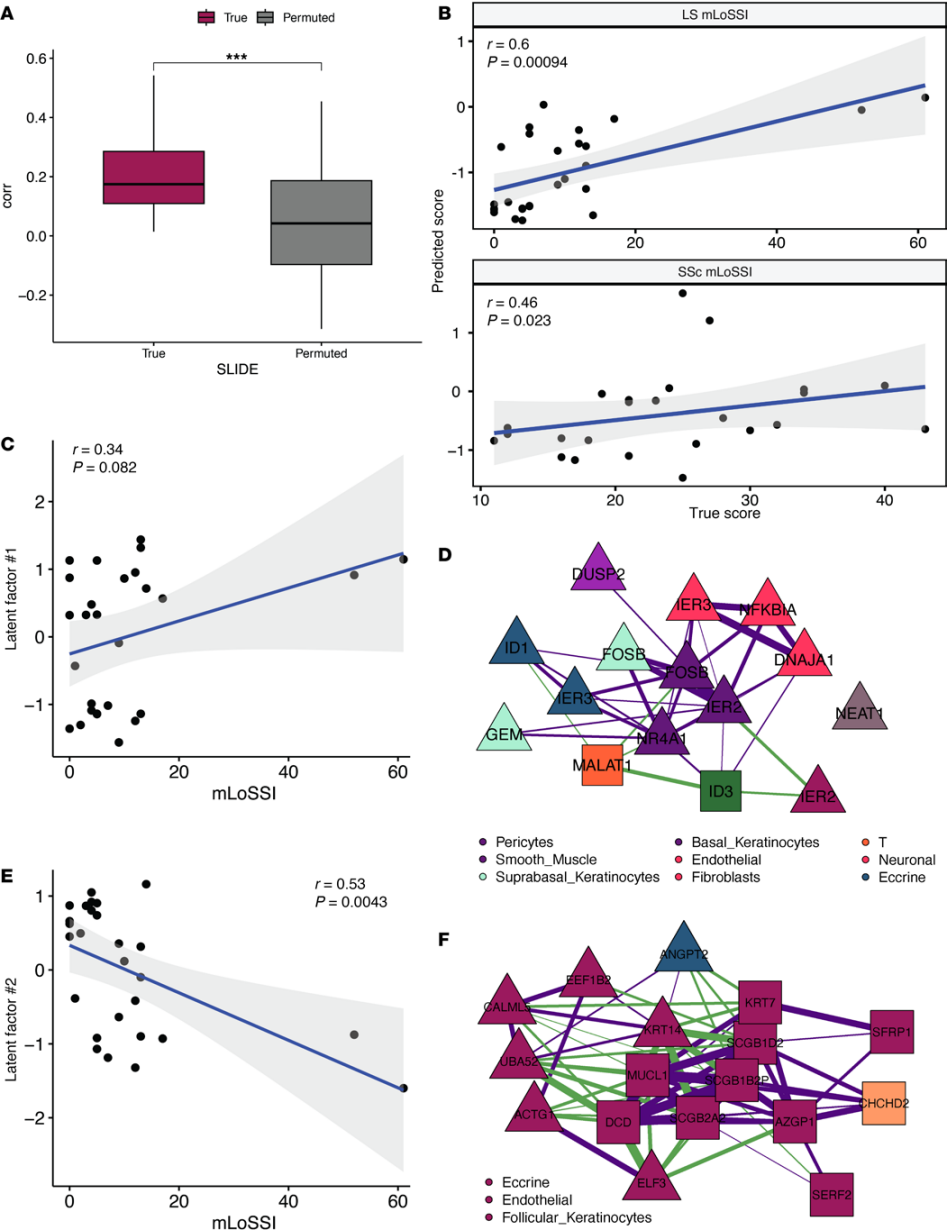
Transcriptomic signatures associated with mLoSSI score in LS. (**A**) SLIDE model performance measured by correlation with mLoSSI using k-fold cross-validation. Asterisks indicate Wilcoxon *P* < 0.001. (**B**) Latent factors trained to predict mLoSSI score in LS (top) can cross-predict MRSS in SSc (bottom). Plot shows true and predicted scores with *P* values measured by Spearman correlation. (**C**) Spearman correlation between latent factor 1 levels and mLoSSI score. (**D**) Correlation network representation of latent factor 1. Purple and green edges indicate positive and negative correlations, respectively. Triangles indicate genes associated with higher mLoSSI, and squares indicate genes associated with lower mLoSSI. (**E**) Spearman correlation between latent factor 2 levels and mLoSSI score. (**F**) Correlation network representation of latent factor 2. Box plots show the interquartile range, median (line), and minimum and maximum (whiskers).

These results encouraged us to evaluate LS from a severity perspective. We wanted to test whether we were able to identify signatures that provide insights into differences across the disease severity spectrum, beyond simply discriminating LS and healthy. So, we then used SLIDE to build a model that provides inference into the severity of LS as quantified by modified Localized Scleroderma Severity Index (mLoSSI) scores, where we regressed out sex-specific effects (25) (Figure 5A). The model identified 5 significant latent factors (Supplemental Table 5).

Considering our success in applying adult LS latent factors in SSc earlier, we evaluated our mLoSSI latent factors applied to the modified Rodnan Skin score (MRSS), an analogous severity score in SSc (26) (Figure 5B). Remarkably, we identified the same factors that could predict mLoSSI score were applicable to predicting MRSS in patients with SSc (21). Together, these results suggest that latent factors that provide inference into differences between adult LS and healthy as well as severity of adult LS generalize to adult SSc. When we investigated our mLoSSI latent factors, we found a combination of cell-extrinsic and cell-intrinsic effects. The cell-extrinsic effects were positively correlated with mLoSSI score (Figure 5C) and consisted of multicellular crosstalk between suprabasal keratinocytes, smooth muscle cells, fibroblasts, and pericytes (Figure 5D). Genes associated with apoptosis (*IER2*, *IER3*) were upregulated across several cell types in this model, including endothelial cells, eccrine cells, suprabasal keratinocytes, and T cells. Finally, we saw a latent factor negatively associated with LS severity (mLoSSI score) (Figure 5E). Gene expression in this module consisted almost entirely of eccrine cells, with small contributions from endothelial cells and follicular keratinocytes with a clear correlation of upregulated *ACTG1* and *KRT14* in eccrine cells associated with higher mLoSSI score, while strong correlations associated with lower mLoSSI score were observed between *CHCHD2* in follicular keratinocytes and *MUC1*, *DCD*, and *KRT7* in eccrine cells (Figure 5F).

### Spatial transcriptomics validation of key latent factors identified by SLIDE.

**Figure 6:**
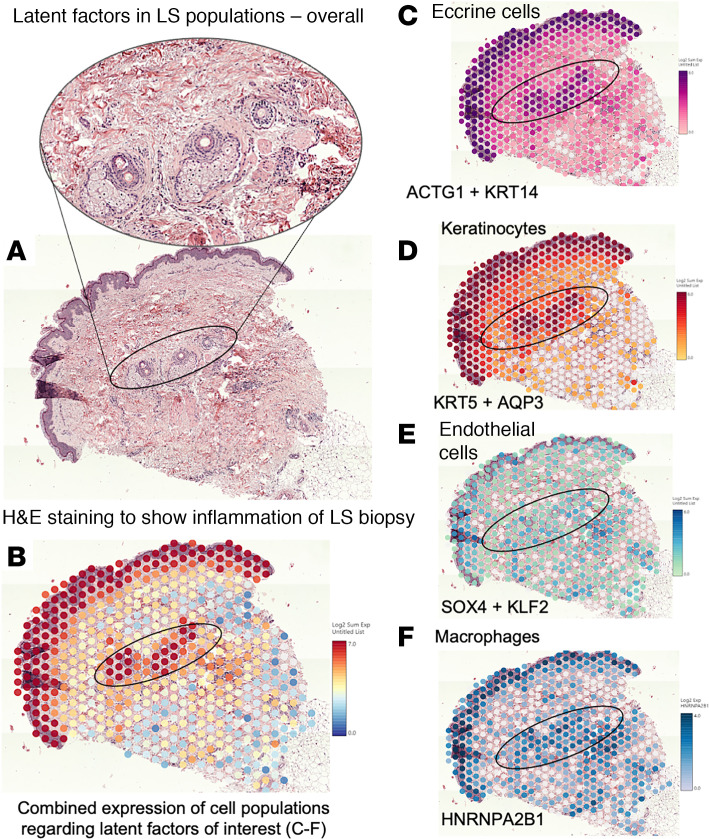
Spatial transcriptomics images of a representative LS from the dataset. (**A**) The H&E stain of the representative LS tissue shown with areas of interest blown out. Marker genes (MUCL1 for eccrine, KRT10 for keratinocytes, PECAM1 for endothelial cells, and CD163 for macrophages) were used to look for latent factors identified in the corresponding cell types. (**B**) Colocalization and intensity of signals from all latent factors around sebaceous glands/follicles with inflammation pointing toward possible interaction. (**C**) KRT14 and ACTG1 upregulated in eccrine cells and (**D**) KRT5 and AQP3 in keratinocytes. These were colocalized in the same areas. (**E**) SOX4 and KLF2 were upregulated in endothelial cells, and (**F**) HNRNPA2B1 was upregulated in macrophages and colocalized in the same area.

We also applied spatial transcriptomics technology (Visium, 10x Genomics, CITE assist) to formalin-fixed, paraffin-embedded (FFPE) sections of skin tissues collected at the same time as that of the sequences and analyzed them to look for cells and genes that were established as important latent factors through SLIDE. Though spatial transcriptomics is ever evolving, this present method to analyze the spatial profile of tissues has a greater and more sensitive single-cell resolution with ability to detect higher number of profiled genes, and using this, we intended to validate the genes identified through SLIDE as important latent factors in overall LS pathogenesis. We used this technology to compare the H&E slides (Figure 6A), then spatial transcriptomics on a representative LS sample from a patient in the study to visualize colocalization of the genes and provide an unbiased picture of spatial composition and skin tissue atlas of the patients with LS (Figure 6B). The gene expression matrix was used to analyze and validate whether the latent factors were interacting and were indeed playing roles in the progression of the disease. We also wanted to identify the location of these signals. *MUCL1* for eccrine cells, *KRT10* for keratinocytes, *PECAM1* for endothelial cells, and *CD163* for macrophages were used as marker genes to identify the corresponding latent factors in the cell types. We could identify *KRT14* and *ACTG1*, which were expressed in eccrine cells (Figure 6C), and *KRT5* and *AQP3* present in the keratinocytes (Figure 6D), as predicted by our SLIDE latent factor analysis model. We observed some overlap in keratinocyte markers within the eccrine tissue region, likely due to the diverse cell types present in this area. Specifically, *KRT14* and *KRT5*, which are typically associated with keratinocytes, were also detected in the eccrine region in our spatial profiling analysis. This finding aligns with prior studies showing that markers like *KRT5*/*KRT14* are expressed in myoepithelial and keratinocyte-like cells within eccrine tissue, as reported by Cui and Schlessinger (27). The location in similar areas revealed interactions between expected cell types (Figure 6F). We also identified endothelial cells with upregulated *SOX4* and *KLF2* (Figure 6E) and macrophages with upregulated *HNRNPA2B1* (Figure 6F) colocalized in the same area with other cell types identified in the latent factors, validating the genes and their corresponding cell types, which were identified by the analysis with stained tissue sections. The areas near the epidermis, which were rich in keratinocytes, showed upregulation of most of the genes and cell types, indicating the interaction between different cells at the site of onset of tissue injury. It was also enriched in the latent factors identified through SLIDE. We also identified areas with inflammation around hair follicles rich in eccrine glands and macrophages shown by H&E, which were also concentrated with these latent factors. All these validated a possible cell-cell interaction in LS with increases in the identified latent factor targets.

## Discussion

### Shared pathology across LS and SSc.

In this study, we used transcriptomic profiles from skin biopsies taken from pediatric and adult patients with LS and identified latent factors that demonstrate how different cell types, intrinsically and extrinsically, may be driving LS pathogenesis. We also identified a shared basis for pathogenesis with SSc (20). Our analyses highlight gene expression modules that are distinct to LS in a variety of contexts associated with age and severity. Broadly, we appreciate that the cell types implicated in LS largely recapitulate pathology in SSc and the surprising finding that keratinocyte activation in adult LS qualitatively resembles SSc in adults more closely than pediatric LS.

In LS, prior bulk RNA-Seq analyses identified upregulated HLA-, IFN-, and TNF-associated genes corresponding to both degree of inflammatory infiltrate and collagen deposition, supporting the dogma that LS is an inflammation-driven fibrotic disease (13). In SSc, studies suggest the severity of skin score corresponds to both macrophage and fibroblast markers, portraying them as important cellular players in the disease progression (28, 29). More recently, keratinocytes have been identified as key contributors to SSc pathogenesis, with studies demonstrating a direct correlation between the upregulation of *KRT6*, *KRT16*, and *KRT14* in the skin from patients with higher MRSS. This dysregulation was also observed in healing wounds and radiation-damaged skin (22, 30). Additionally, KRT16 and KRT17 have been shown to be elevated in other inflammatory skin diseases, such as psoriasis and atopic dermatitis, when compared with healthy controls (31). In our recent study using the SLIDE framework on scRNA-Seq data from patients with SSc, we uncovered significant latent factors, revealing altered transcriptomic states in myeloid and fibroblast cells with a unique keratinocyte-centric signature (21).

Applying SLIDE to the full LS data set (pediatric and adult), we identified similarities between the involvement of key cell types in LS and SSc. Our analysis in LS also provides insight into how immune effectors can drive pathology in coordination with matrix-remodeling nonimmune cells. For example, enhanced expression of *KRT5*, along with upregulation of *JUN*, *FOS*, and *JUND* expression in suprabasal keratinocytes across LS samples, suggests that these cells contribute to fibrotic tissue damage through mechanisms similar to those observed in SSc (21). Some of these proteins (JUN and JUNB) have been shown to be elevated in other skin diseases, like pro-fibrotic Schwann cell development, by immunohistological staining (32). These findings, paired with *HLAE* in macrophages and upregulation of *KLF2* and *SOX4* in endothelial cells, factors similarly increased in other inflammatory skin diseases such as atopic dermatitis, may indicate the presence of a cell-extrinsic pro-inflammatory circuit in both pediatric and adult LS. Additionally, the upregulation of *NEAT1*, a known regulator of cell migration and apoptosis, suggests suppressed proliferation via TGF-β–dependent pathways may be an important driver of activated fibroblasts and fibrosis in LS. Notably, the serum levels of long noncoding RNAs (lncRNAs) of NEAT1 and MALAT1 have been found to be elevated in patients with Behçet’s disease, which shares a deep dermal infiltrate with LS, suggesting that these lncRNAs may play a similar role in the pathogenesis of LS (33). Fibroblasts also have correlated gene expression with macrophages via upregulation of *HNRNPA2B1*, which regulates IFN-γ signaling, supporting our recent findings of a strong type II IFN communication exchange between macrophages and fibroblasts in LS (17, 34).

### Adult LS and SSc share cell-intrinsic and cell-extrinsic signatures of pathogenesis.

Our overall model suggests there is age-specific heterogeneity in our cohort, so we examined latent factors separately in adult and pediatric LS models. In alignment with previously published SSc findings, our adult LS model indicates LS pathogenesis involves keratinocyte and macrophage interaction along with some extrinsic interaction with B/mast cells. Keratinocytes have been understudied as a cell type in LS, though they likely play important roles as sensors and respond to stimuli like proliferation, apoptosis, and other inflammatory signals during the pathogenic process (35). Changes in the normal functioning of keratinocytes have been shown to increase inflammatory responses in skin during the scleroderma process (36). In our LS samples, keratinocytes produce transcription factors *JUN/FOS*, which help in the activation of cytokines and chemokines, further aiding macrophage migration to the site of inflammation. The upregulation of *KRT2* and *AQP3* observed in the suprabasal and basal keratinocytes, respectively, likely play important roles in the fibrotic disease progression. *AQP3* has been implicated in TGF-β–induced fibrosis and is upregulated during postinflammatory wound healing, contributing to fibrotic tissue changes (37). Additionally, *AQP3* is elevated in chronic inflammatory skin diseases such as atopic dermatitis, where it may contribute to disease pathogenesis (38). Similarly, *KRT2* is known to play a role in the fibrotic process, with genetic studies suggesting its utility as a marker for disease progression in SSc (39). Basal keratinocytes also demonstrated an upregulation of *MALAT1* and strong communication with fibroblasts in the model. *MALAT1* has been typically known to be a result of *TGFB*-dependent upregulation of chemokines and cytokines and has been directly implicated to be an active factor in fibrosis (40, 41). NEAT1 has been shown to be an important noncoding RNA present as a responsible factor in a number of fibrotic disorders as well (42). We suspect the migrated inflammatory macrophages producing *CXCL8* in our LS model (more strongly in adult LS) support a crucial role in response to tissue injury. This is on par with findings in adult SSc describing *CXCL8* as a convincing biomarker in both skin and lung disease; being expressed by dermal and alveolar macrophages; and corresponding with circulating peripheral blood levels, degree of fibrosis, and disease severity (43, 44). Furthermore, studies in inflammatory skin-related autoimmune diseases, including psoriasis and Behçet’s, also report upregulation of *CXCL8* using immunohistology, reinforcing its role in inflammation and tissue injury (45). Upregulation of *SRGN* and *CLIC1* is known to stimulate endothelial injury, vascular inflammation, and stress and is expressed by B and mast cell types in the model, possibly due to *CXCL8* activity. The adult LS model also supports upregulation of *S100A4* in B cells, a damage-associated molecule pattern molecule, which is elevated at sites of inflammation. This molecule has also been demonstrated to be upregulated in the dermis of psoriatic skin compared with normal skin (46). S100A4 is known to increase during stress and tissue damage caused by sustained inflammation and acts through receptors like TLR4 and RAGE to increase extracellular matrix and myofibroblast formation, leading to fibrosis (47).

Endothelial cells also demonstrated an influence in the cell-extrinsic communication in the adult LS model. *CLDN5*, an important tight junction protein that has been associated with high rigidity and loss of elasticity in cells leading to skin tightening (48), and *CLU*, a biomarker of lung fibrosis in chronic obstructive pulmonary disease (49), were both upregulated in adult LS endothelial cells. *CLU* and *CLDN5* had strong correlations with each other but also with B/mast cells and macrophages.

### The pediatric LS signature reflects higher inflammatory signaling than the adult LS signature.

In pediatric LS, we observe a greater level of interaction between smooth muscle cells, fibroblasts, and T cells, contrasting the keratinocyte- and macrophage-dominant interactions in adults. Notably, *FLIPL1* and *DUSP1* are upregulated in smooth muscle cells and interact with T cells. In our previous analyses of peripheral blood and skin samples from pediatric patients with LS, T cells emerged as a predominant feature, particularly during active disease and showing correlation with the mLoSSI score. Specifically, a Th1-type phenotype predominates and is associated with a higher degree of inflammation and fibrosis (12, 13, 50).

*S100A11*, a gene associated with uncontrolled cellular proliferation in some cancers (51), was upregulated in fibroblasts along with *NEAT1* in the pediatric LS model. They are all positively correlated with each other and may be important in the fibrotic transition between cells after the initial inflammatory onset of the disease observed in the pediatric population. Fibroblasts also have a cell-extrinsic relationship with T cells where *SFN* (52) and *DUSP2* (53) are upregulated in T cells, both of which have been shown to be major players in onset of autoimmune diseases and may be used as markers for detection of some autoimmune disorders, especially rheumatoid arthritis, where suppression of *DUSP2* has been shown to have a positive outcome on the progression of the disease (54).

### Inference of multivariate signatures of pathogenesis enabled by interpretable machine learning.

Surprisingly, the latent factors identified in our disease severity model can similarly predict severity in SSc using MRSS without having adjusted MRSS for sex-specific bias. These subjective measures of severity are relatively consistent across practitioners. We adapt physician scoring for severity (mLoSSI) to identify transcriptomic modules that describe the spectrum of LS pathology. We utilize the mLoSSI score, a composite measure of cutaneous activity (55) at different regions of the body, by first normalizing for sex-specific bias in autoimmune prevalence and performing regression using our calculated latent factors. Notably, we identify a role for eccrine cells in LS pathology, and communication with follicular keratinocytes through *CHCHD2*, as well as reiterate the involvement of suprabasal keratinocytes we saw in our previous models. A previous study using mathematical models on single-cell data showed that KRT6A and S100A8 proteins in keratinocytes correlate with increased skin score severity in SSc (20). In our similar LS analysis, we found an upregulation in *ACTG1* and *KRT14* in eccrine cells in LS across all ages. These genes were associated with higher mLoSSI scores and are known to play roles in actin-mediated cytoskeleton formation. Additionally, they have previously been utilized as biomarkers for lung fibrosis. Both these were often found to be upregulated during the fibrotic stage of skin-related diseases.

Our present analysis is limited to inference from transcriptomic data, as they were the only data available from our cohort; our analysis and inference would likely improve with proteomic or chromatin accessibility data. Nonetheless, using a recently developed interpretable latent machine learning approach, we identify transcriptomic signatures that underlie the severity of fibrotic destruction. The SLIDE latent factors move beyond biomarkers to putative causal factors that would need to be validated in downstream experiments. SLIDE has been successfully used in a range of contexts across infectious and autoimmune diseases to infer putative mechanisms (21, 56–58). Our latent factors highlight the importance of multivariate analysis in describing these heterogeneous diseases, as traditional univariate analyses are often underpowered and simply provide correlative insights but not actual inference of the basis of pathophysiology. SLIDE’s unique properties help us converge on actual mechanistic insights. Overall, this motivates the use of multivariate learning approaches that provide inference beyond prediction in similar contexts.

## Methods

### Sex as a biological variable.

The sex distribution in this study is as follows: Out of a cohort of *n* = 44 total samples, there are 27 patients with LS and 17 controls; within LS, there are 17 female individuals and 10 male individuals; within controls, there are 10 female individuals and 7 male individuals. This distribution roughly reflects the true disease burden with higher incidence in female individuals.

### Human patient skin samples.

LS skin biopsies were obtained as 4 mm punches of affected areas from research participants in the National Registry of Childhood Onset Scleroderma (NRCOS) (University of Pittsburgh, PRO11060222), Connective Tissue Disease (CTD) Registry (University of Pittsburgh, PRO19090054), and Morphea in Adults and Children (MAC) Registry (University of Texas Southwestern, STU112010-028) cohorts. Healthy controls were taken from tissue discard IRB (University of Pittsburgh, STU19070023) and were age- and sex-matched. Patients enrolled with LS all met the Padua diagnostic criteria for LS (59) and were classified as having pediatric-onset disease if symptoms began before age 18 or adult-onset disease if symptoms began at age 18 or older. Disease severity was scored among the patients using the Localized Scleroderma Cutaneous Assessment Tool (55, 60), specifically with focus on the disease activity subcomponent, the mLoSSI (25).

### scRNA-Seq and data preprocessing.

We analyzed the transcriptomes from 44 skin biopsies comprising a diverse cohort of LS and control samples. This cohort includes adult and pediatric samples from LS patients with a variety of subtypes along with age-matched controls (see Supplemental Table 1). Samples were processed with 10x Genomics Chromium instrument as described in our earlier publication, with approximately 2,600–4,300 cells loaded per dissociated skin sample, followed by library preparations and single-cell sequencing using the Illumina NextSeq 500 platform (Figure 1A) (17, 61).

After sequencing, FASTQ files were processed with Cell Ranger and then analyzed with Seurat. Cell Ranger was used to perform alignment, filtering, and unique molecular identifier counting through the *count* function and outputs the gene-barcode matrices. This function also used reference human genome GRCh38 for mapping. These resulting h5 files were then aggregated using the Cell Ranger *aggr* function to get the compiled matrix of all relevant samples.

Data analyses of the matrices generated were performed using R (version 4.3.0), specifically the Seurat 4.3.0 package for normalization of gene expression and identification and visualization of cell populations (total cells, 125,998). Our data were filtered with parameters of nFeature_RNA cutoffs of below 5,500 and above 200, percentage mitochondrial content below 25%, and percentage ribosomal content below 55%. Principal component analysis was performed on the highly variable genes, and the Harmony (62) package was used to integrate the dataset, removing variation per sample (library_id). Cells were then clustered using a smart local moving algorithm (63), and these results underwent dimension reduction and visualized by UMAP (64). AddModuleScore was utilized to calculate the average expression levels of each input (either gene or cluster) on a single-cell level. Known marker genes were used to identify different cell types and annotated accordingly.

### Spatial transcriptomics.

Visium Spatial Gene Expression (10x Genomics) was run on the FFPE skin biopsy taken during the same time as the biopsy for the scRNA-Seq from an adjacent affected area of a patient with LS. Five-micrometer sections were taken from these FFPE blocks and stained with H&E (Hematoxylin catalog 7211/7212 and Eosin-Y [Alcoholic] catalog 7111L) (Thermo Fisher Scientific Richard-Allan Scientific Signature Series Stains protocol), followed by the Visium CytAssist protocol for FFPE tissues on a slide cassette with 6.5 × 6.5 mm capture area (65). The sequencing data and the H&E-stained image were merged and aligned by the spatial coordinate of the slide, and Loupe browser was used to study images and visualize gene expression (66).

### SLIDE to determine gene expression modules associated with LS.

We determined 32 cell type–specific clusters present in our samples to identify cell types present in the skin (epidermis and dermis), including several immune and nonimmune/stromal cells prevalent in LS, such as T cells and macrophages and several types of keratinocytes, respectively, and identified the top 50 high-variance genes in each cell type, giving us a total of 1,550 cell type–specific genes to use for SLIDE. This cluster-gene expression matrix was decomposed into overlapping modules of genes (latent factors) that were regressed to the LS response of interest. After model formulation, we identified the latent factors most important for inference by using Gaussian knockoffs (67) to control for an FDR of 0.05. We determined that our latent factors detected biologically relevant signals, and not spurious correlation structure in the data, by testing latent factor predictions on shuffled labels using a rigorous k-fold cross-validation framework, consisting of repeated trials training the model on a subset of the data (training set) and evaluating its performance on a different subset of the data (testing set). We used SLIDE to identify features that separate LS and healthy samples, as well as features that define age-specific LS or predict LS severity. Within these latent factors, we visualize the features using the top 10 genes by contributions to latent factor composition and the top 10 predictive of LS label. Thus, the latent factors we identified represent broadly important expression modules that differ between healthy and affected patients at baseline (20, 21).

### Correlation networks for cell type–specific molecular signatures.

Correlation networks were plotted to show the correlations between top features (described above) in each latent factor. We denoted cell type using color and shape to indicate a feature’s association with the response of interest. We visualized correlations with an absolute value above 0.25 and used purple edges for positive correlations and green edges for negative correlations (Figure 2, C and F; Figure 3, D and F; Figure 4, C and E; and Figure 5, D and F).

### Cross-prediction.

Latent factors from SLIDE were applied to an scRNA-Seq data set from 24 adult patients with SSc at the adult center at University of Pittsburgh (21) to validate the cross-predictive performance of our findings in an SSc cohort. In short, we used the weight matrix that defines the composition of our SLIDE latent factors to calculate sample loadings for unseen expression data and evaluated predicted versus actual response using the same linear model coefficients. To apply our latent factors to the SSc data, we first used the Seurat functions FindTransferAnchors and TransferData to assign cells in the SSc data to the same cluster space we used for LS. Next, we calculated pseudobulked expression values for each cluster as described above and subset this data to have the same cluster genes as our original data; any missing features in our new data were then subsequently removed from our original data so that both data were evaluated using the same cluster genes. We then decomposed this expression matrix using our defined weight matrix to evaluate latent factor performance in predicting response for the unseen dataset. For our classification cross-predictions, we evaluated our original and new data using feature-matched latent factors. For our regression cross-predictions, we used identical parameters, reran SLIDE on the feature-matched original data, and applied these latent factors to predicting MRSS in SSc. The latent factors were largely preserved and had a cosine similarity greater than 0.98 with their analogs in the second round of SLIDE.

### Statistics.

We used a multivariate machine learning approach, SLIDE (with an FDR threshold of 0.05), to establish latent factor significance. All relevant details are provided in the above sections (SLIDE) to determine gene expression modules associated with LS, correlation networks for cell type–specific molecular signatures, and cross-prediction. *P* < 0.05 was considered statistically significant.

### Study approval.

Patients and/or the public were not involved in the design, conduct, reporting, or dissemination of this research. Patient consent for publication is not applicable.

LS skin biopsies were obtained as 4 mm punches from research participants in the NRCOS (University of Pittsburgh PRO11060222), CTD Registry (University of Pittsburgh, PRO19090054), and MAC Registry (University of Texas Southwestern, STU112010-028) cohorts. Healthy controls were taken from tissue discard IRB (University of Pittsburgh, STU19070023) and were age- and sex-matched.

### Data availability.

Data are available at NCBI GEO GSE264508, GSE138669, and GSE288490. All code, sample metadata, and documentation are available at https://github.com/jishnu-lab/LS_pathogenesis (commit ID 92109a2). Values for all data points found in graphs are in the Supporting Data Values file.

## Author contributions

JD and KST conceived of the study and supervised all aspects. KST came up with the experimental design. JD designed all computational analyses. HJ and RL helped with data acquisition. ABIR carried out all computational analyses with help from JSB. AS, TH, GW, and TT carried out the experiments. ABIR, AS, JD, and KST wrote the manuscript with inputs from all authors. Authorship order was determined based on equal contributions to the experimental design, data collection, analysis, and manuscript preparation, with co–first authors designated to acknowledge their equivalent intellectual contributions to this work.

## Supplementary Material

Supplemental data

Supporting data values

## Version 1 04/08/2025

Electronic publication
