# Supplementary material for: Unique and shared transcriptomic signatures underlying localized scleroderma pathogenesis identified using interpretable machine learning
Source: JCI Insight. 2025 Apr 8;10(7):e185758. doi: 10.1172/jci.insight.185758 (PMC11981619; doi:10.1172/jci.insight.185758)
Supplement: Supplemental data [file jciinsight-10-185758-s170.pdf]

Supplemental Figure 1: Single cell RNA sequencing defines 32 total cell populations in the skin of patients with localized scleroderma compared with age and sex-matched healthy controls. A) The initial UMAP of 32 clusters including cell type name is displayed. We later consolidated these clusters in 15 for easier analyses and cellular relations. For example, clusters 7, 8, 9, 11, 26, and 31 are all Endothelial cells sharing core common annotation markers and were labeled collectively as Endothelial cells (*Figure B here*), population 6 in the final clustering, main Figure 1. **B)** Both LS and healthy control subjects have cells populating all cell types, supporting no major sample type skewing. **C)** The cell type composition for the healthy and LS subjects demonstrates higher proportions of T cells, keratinocytes, and endothelial cells, while healthy subjects have more fibroblasts.

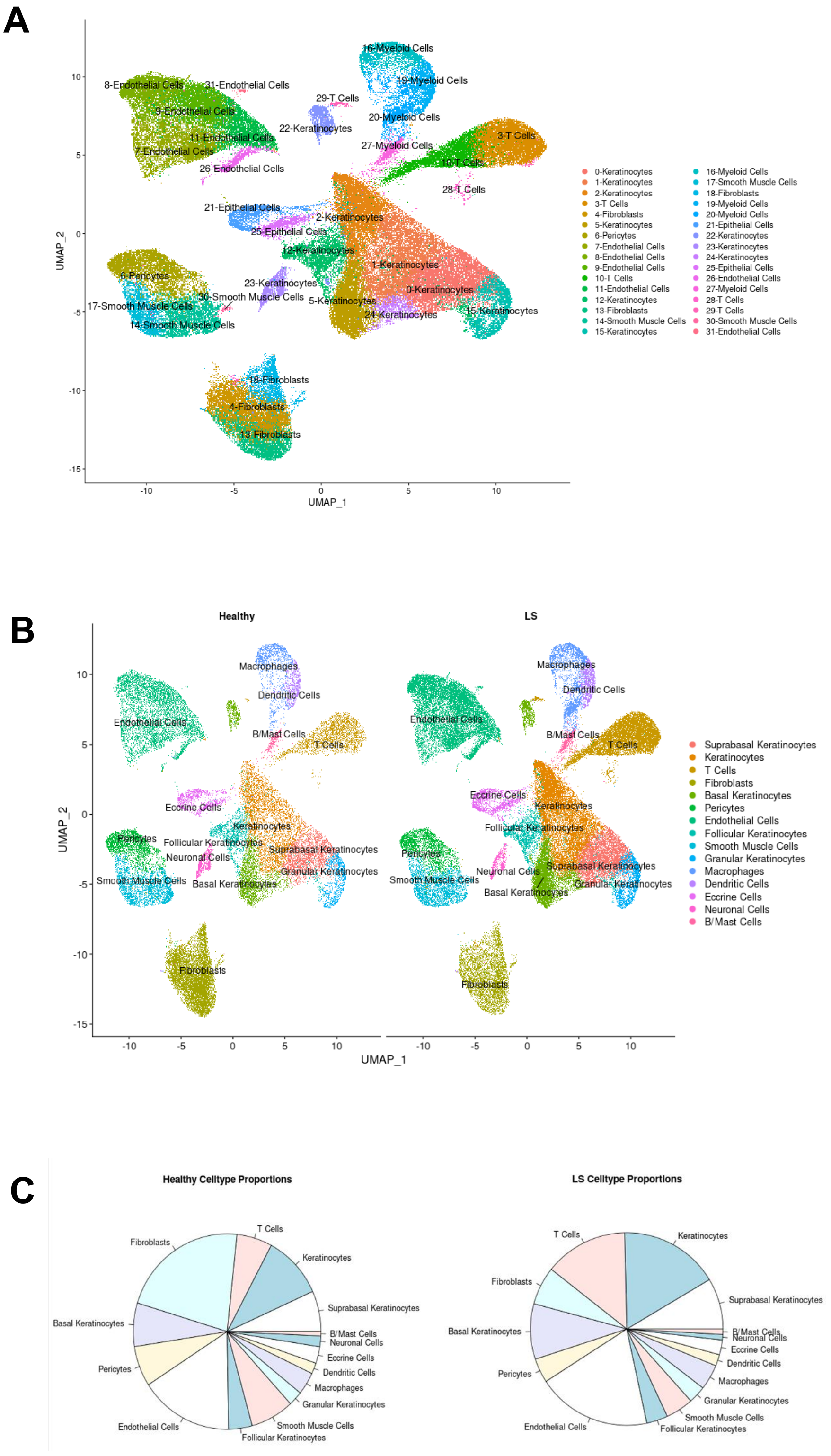
A
UMAP_1
UMAP_2
0-Keratinocytes
1-Keratinocytes
2-Keratinocytes
3-T Cells
4-Fibroblasts
5-Keratinocytes
6-Pericytes
7-Endothelial Cells
8-Endothelial Cells
9-Endothelial Cells
10-T Cells
11-Endothelial Cells
12-Keratinocytes
13-Fibroblasts
14-Smooth Muscle Cells
15-Keratinocytes
16-Myeloid Cells
17-Smooth Muscle Cells
18-Fibroblasts
19-Myeloid Cells
20-Myeloid Cells
21-Epithelial Cells
22-Keratinocytes
23-Keratinocytes
24-Keratinocytes
25-Epithelial Cells
26-Endothelial Cells
27-Myeloid Cells
28-T Cells
29-T Cells
30-Smooth Muscle Cells
31-Endothelial Cells
B
Healthy
LS
UMAP_1
UMAP_2
Suprabasal Keratinocytes
Keratinocytes
T Cells
Fibroblasts
Basal Keratinocytes
Pericytes
Endothelial Cells
Follicular Keratinocytes
Smooth Muscle Cells
Granular Keratinocytes
Macrophages
Dendritic Cells
Eccrine Cells
Neuronal Cells
B/Mast Cells
C
Healthy Celltype Proportions
LS Celltype Proportions
Fibroblasts
T Cells
Keratinocytes
Suprabasal Keratinocytes
B/Mast Cells
Neuronal Cells
Eccrine Cells
Dendritic Cells
Macrophages
Granular Keratinocytes
Smooth Muscle Cells
Follicular Keratinocytes
Endothelial Cells
Pericytes
Basal Keratinocytes

Supplementary Table 1: Demographic information for LS cohort and controls. Pediatric and adult LS cohort with age matched controls. Categories are defined as 1A = adult healthy control; 1B = pediatric healthy control; 2A = adult LS; 2B = pediatric LS

Category	NRCOS	Sample ID	Chemistry	Sample Type	Bx Location	Onset	Age of onset (Yr)	Disease Duration (Yr)	Age at biopsy (Yr)	Gender	Race/Ethnicity	Active/Inactive	Subtype	Antibody Status	LoSAI/mLoSSI	LoSDI	PGA-A	PGA-D	PGA-S
1A	adult control	SC50	V2	Fresh	Forearm	Adult	N/A	N/A	64	M	Caucasian	N/A	Healthy	N/A	N/A	N/A	N/A	N/A	N/A
1A	adult control	SC68	V2	Fresh	Forearm	Adult	N/A	N/A	48	F	Caucasian	N/A	Healthy	N/A	N/A	N/A	N/A	N/A	N/A
1A	adult control	SC124	V2	Fresh	Forearm	Adult	N/A	N/A	54	M	Caucasian	N/A	Healthy	N/A	N/A	N/A	N/A	N/A	N/A
1A	adult control	SC125	V2	Fresh	Forearm	Adult	N/A	N/A	61	M	African American	N/A	Healthy	N/A	N/A	N/A	N/A	N/A	N/A
1A	adult control	SC1	V1	Fresh	Forearm	Adult	N/A	N/A	63	M	Caucasian	N/A	Healthy	N/A	N/A	N/A	N/A	N/A	N/A
1A	adult control	SC4	V1	Fresh	Forearm	Adult	N/A	N/A	63	M	Caucasian	N/A	Healthy	N/A	N/A	N/A	N/A	N/A	N/A
1A	adult control	SC18	V1	Fresh	Forearm	Adult	N/A	N/A	66	F	Caucasian	N/A	Healthy	N/A	N/A	N/A	N/A	N/A	N/A
1A	adult control	SC33	V1	Fresh	Forearm	Adult	N/A	N/A	62	F	Caucasian	N/A	Healthy	N/A	N/A	N/A	N/A	N/A	N/A
1A	HSK-060	SC407	V3	Cryostor	Breast	Adult	N/A	N/A	18	F	Caucasian	N/A	Healthy	N/A	N/A	N/A	N/A	N/A	N/A
1A	1223	SC392	V3	Fresh	Forearm	Adult	N/A	N/A	23	M	Caucasian	N/A	Healthy	N/A	N/A	N/A	N/A	N/A	N/A
1A	HSK-056	SC386	V3	Fresh	Breast	Adult	N/A	N/A	21	F	Caucasian	N/A	Healthy	N/A	N/A	N/A	N/A	N/A	N/A
1B	ped control	SC32	V1	Cryostor	Forearm	Peds	N/A	N/A	23	F	Asian	N/A	Healthy	N/A	N/A	N/A	N/A	N/A	N/A
1B	ped control	SC296	V2	Cryostor	Scalp	Peds	N/A	N/A	5	M	Caucasian	N/A	Healthy	N/A	N/A	N/A	N/A	N/A	N/A
1B	ped control	SC297	V2	Cryostor	Midarm	Peds	N/A	N/A	14	F	Hispanic	N/A	Healthy	N/A	N/A	N/A	N/A	N/A	N/A
1B	ped control	HSK053	V2	Fresh	Breast	Peds	N/A	N/A	18	F	African American/ Caucasian	N/A	Healthy	N/A	N/A	N/A	N/A	N/A	N/A
1B	ped control	HSK054	V2	Fresh	Breast	Peds	N/A	N/A	17	F	African American	N/A	Healthy	N/A	N/A	N/A	N/A	N/A	N/A
1B	ped control	PHC003	V2	Cryostor	Midarm	Peds	N/A	N/A	13	F	Hispanic	N/A	Healthy	N/A	N/A	N/A	N/A	N/A	N/A
2A	HJ875	SC445	V3	Cryostor	Abdomen	Adult	27	1	27	F	African American	A	Linear	-	5	2	21	14	-
2A	HJ534	SC446	V3	Cryostor	Abdomen	Adult	57	8	65	M	Caucasian	A	Generalized	-	13	17	32	8	-
2A	HJ 696 (UTSW 5)	SC275	V2	Cryostor	Left flank	Adult	63	2	65	F	Caucasian	A	Generalized, Isomorphic morphea	unknown	5	32	10	23	-
2A	UTSW1-DSK001	SC222AC	V2	Affected Cryostor	Left abdomen	Adult	-	-	65	M	Caucasian	A/I	Generalized	unknown	14	38	18	18	-
2A	1000	SC260	V2	Fresh	Lower back	Adult	34	13	43	M	Caucasian	I	Circumscribed/ Plaque	unknown	0	5	0	18	12
2A	UTSW6	HJ809	V2	Cryostor	Abdomen	Adult	64	1	65	F	Caucasian	A	Generalized	unknown	61	28	90	60	-
2A	UTSW7	HJ867	V2	Cryostor	Right Arm	Adult	54	8	61	F	Caucasian	A	Generalized	unknown	13	39	27	30	-
2A	HJ740	SC408	V3	Cryostor	Abdomen	Adult	62	4	66	F	Caucasian	A	Generalized	-	5	26	4	17	-
2A	HJ861	SC409	V3	Cryostor	Right thigh	Adult	47	0.33	47	F	African American	A	Indeterminate	-	12	17	19	12	-
2A	HJ825/345	SC389	V3	Cryostor	Abdomen	Adult	61	10.8	71	F	Hispanic	A	Linear	-	5	13	8	12	-
2A	HJ 771 (UTSW2)	SC266	V2	Cryostor	Abdomen	Adult	24	13	37	M	Caucasian	A	Linear	unknown	4	34	13	55	-
2A	HJ 773 (UTSW3)	SC267	V2	Cryostor	Left abdomen	Adult	61	2.7	64	F	Caucasian	A	Generalized	unknown	9	14	23	12	-
2A	HJ 656 (UTSW 4)	SC272	V2	Cryostor	Left abdomen	Adult	34	9	43	F	Hispanic	A	Linear	unknown	10	10	23	15	-
2B	474	SC126	V2	Fresh	Upper thigh	Peds	6.3	1.75	8	F	Caucasian	A	Linear	ANA negative, Histone negative, ssDNA negative	17	14	76	35	43
2B	488	SC198	V2	Fresh	Midarm	Peds	13	1.16	14	F	Asian	I	Linear	unknown	0	8	0	18	19
2B	507	SC246	V2	Fresh	Thigh	Peds	12.6	1.15	15	F	Asian	A	Linear	ANA positive (1:160 homogenous), Histone+ ssDNA+	13	12	62	37	47
2B	494	SC259	V2	Fresh	Scalp/forehead	Peds	7.4	8.3	16	M	Caucasian	A	Linear Face	6/2019: Histone negative, ssDNA negative	4	4	29	41	45
2B	414	SC144	V2	Fresh	Lower back	Peds	16.6	3.33	20	M	Caucasian	I	Circumscribed	not done at this visit	7	14	0	52	53
2B	555	SC300	V2	Fresh	Scalp/forehead	Peds	4.8	14.4	20	M	Caucasian	A	Linear Face	not done at this visit	3	5	8	47	52
2B	466	SC424	V3	Cryostor	Scalp and Temple	Peds	10	7	17	F	Caucasian	A	Linear Face	negative ANA	0	3	5	51	53
2B	660	SC442	V3	Fresh	Left Calf	Peds	5	8	13	F	Caucasian	A	Linear	not done at this visit	1	10	33	36	38
2B	662	SC443	V3	Fresh	Left buttocks	Peds	13	3	16	F	Caucasian	A	Circumscribed	Positive ANA (homogenous, negative dsDNA, negative RNP)	4	5	46	22	29
2B	669	SC457	V3	Fresh	Arm	Peds	4	1	5	F	Caucasian	A	Pan sclerotic	not done at this visit	52	68	78	81	95
2B	595	SC361	V2	Cryostor	Right Thigh	Peds	10	0.76	11	F	Caucasian	A	Linear	ANA negative, Histone negative	9	6	65	20	42
2B	409	SC388	V3	Cryostor	Upper Back	Peds	10	6.8	16	F	Caucasian	I	Circumscribed	Histone negative	0	4	0	20	18
2B	437	SC391	V3	Fresh	Right thigh	Peds	14	7	21	F	Caucasian	A	Linear	ANA positive (homogenous), histone negative	12	8	35	30	44
2B	673	SC455	V3	Cryostor	Thigh	Peds	11	1.3	13	F	Caucasian	A	Linear	Positive Histone, negative PM1	2	5	14	42	40

Supplemental Table 2: Additional SLIDE latent factors from model used to classify LS and healthy patients. Additional latent factors chosen by SLIDE for classifying LS and Healthy samples from combined pediatric and adult samples. Latent factor number is used solely to indicate gene membership and does not correspond to any ranking.

Gene	Association	latent factor number	model
**C.0.LGALS7**	Higher in Healthy	2	LS vs H
**C.22.LGALS7**	Higher in Healthy	2	LS vs H
**C.27.CD44**	Higher in Healthy	2	LS vs H
**C.12.HOPX**	Higher in Healthy	2	LS vs H
**C.10.NEAT1**	Higher in LS	2	LS vs H
**C.23.S100A4**	Higher in Healthy	3	LS vs H
**C.23.CRYAB**	Higher in Healthy	3	LS vs H
**C.23.SPARC**	Higher in Healthy	3	LS vs H
**C.23.PRNP**	Higher in Healthy	3	LS vs H
**C.23.TUBA1A**	Higher in Healthy	3	LS vs H
**C.23.GSN**	Higher in Healthy	3	LS vs H
**C.23.TUBA1B**	Higher in Healthy	3	LS vs H
**C.23.PSAP**	Higher in Healthy	3	LS vs H
**C.20.HLAC**	Higher in LS	3	LS vs H
**C.23.ZFP36L1**	Higher in LS	3	LS vs H
**C.14.MFAP4**	Higher in Healthy	3	LS vs H

Supplemental Table 3: Additional SLIDE latent factors from model used to classify adult LS and adult healthy patients. Additional latent factors chosen by SLIDE for classifying LS and Healthy samples from adult samples. Latent factor number is used solely to indicate gene membership and does not correspond to any ranking.

Gene	Association	latent factor number	model
**C.4.COL3A1**	Higher in LS	1	Adult LS vs H
**C.13.COL3A1**	Higher in LS	1	Adult LS vs H
**C.13.COL1A1**	Higher in LS	1	Adult LS vs H
**C.18.COL6A1**	Higher in LS	1	Adult LS vs H
**C.1.AQP3**	Higher in LS	1	Adult LS vs H
**C.26.PLAC9**	Higher in LS	1	Adult LS vs H
**C.17.NFKBIA**	Higher in Healthy	1	Adult LS vs H
**C.27.DDX5**	Higher in LS	1	Adult LS vs H
**C.4.NFKBIA**	Higher in Healthy	1	Adult LS vs H
**C.23.FOS**	Higher in Healthy	2	Adult LS vs H
**C.23.JUN**	Higher in Healthy	2	Adult LS vs H
**C.23.JUNB**	Higher in Healthy	2	Adult LS vs H
**C.23.HES1**	Higher in Healthy	2	Adult LS vs H
**C.18.CIRBP**	Higher in LS	2	Adult LS vs H
**C.3.SLC2A3**	Higher in Healthy	2	Adult LS vs H
**C.23.SKP1**	Higher in Healthy	2	Adult LS vs H
**C.4.TCF4**	Higher in LS	3	Adult LS vs H
**C.23.EMP3**	Higher in Healthy	3	Adult LS vs H
**C.25.UQCRH**	Higher in LS	3	Adult LS vs H
**C.25.EEF1B2**	Higher in LS	3	Adult LS vs H
**C.25.HNRNPDL**	Higher in LS	3	Adult LS vs H
**C.25.SNHG8**	Higher in LS	3	Adult LS vs H
**C.25.HINT1**	Higher in LS	3	Adult LS vs H
**C.25.S100A6**	Higher in LS	3	Adult LS vs H
**C.25.PERP**	Higher in LS	3	Adult LS vs H
**C.20.COX7C**	Higher in LS	3	Adult LS vs H
**C.20.EEF1B2**	Higher in LS	3	Adult LS vs H

Supplemental Table 4: Additional SLIDE latent factors from model used to classify adult and pediatric LS patients. Additional latent factors chosen by SLIDE for classifying Pediatric and Adult samples in the LS cohort. Latent factor number is used solely to indicate gene membership and does not correspond to any ranking.

Gene	Association	latent factor number	model
**C.7.HLADRB5**	Higher in Peds LS	1	LS onset
**C.8.HLADRB5**	Higher in Peds LS	1	LS onset
**C.9.HLADRB5**	Higher in Peds LS	1	LS onset
**C.11.HLADRB1**	Higher in Adult LS	1	LS onset
**C.20.DDX5**	Higher in Peds LS	1	LS onset
**C.3.CST7**	Higher in Adult LS	1	LS onset
**C.9.CTGF**	Higher in Adult LS	1	LS onset
**C.10.DNAJA1**	Higher in Peds LS	1	LS onset
**C.5.AREG**	Higher in Peds LS	2	LS onset
**C.14.RHOB**	Higher in Peds LS	2	LS onset
**C.17.ZNF331**	Higher in Peds LS	2	LS onset
**C.17.AVPR1A**	Higher in Peds LS	2	LS onset
**C.17.CREM**	Higher in Peds LS	2	LS onset
**C.17.NR4A2**	Higher in Peds LS	2	LS onset
**C.17.MAT2A**	Higher in Peds LS	2	LS onset
**C.17.CYCS**	Higher in Peds LS	2	LS onset
**C.17.EIF4A3**	Higher in Peds LS	2	LS onset
**C.20.EEF1B2**	Higher in Peds LS	3	LS onset
**C.20.EEF1D**	Higher in Adult LS	3	LS onset
**C.20.CYBA**	Higher in Adult LS	3	LS onset
**C.20.COX7C**	Higher in Adult LS	3	LS onset
**C.20.ZFAS1**	Higher in Adult LS	3	LS onset
**C.20.TOMM7**	Higher in Adult LS	3	LS onset
**C.20.HNRNPA1**	Higher in Adult LS	3	LS onset
**C.20.RACK1**	Higher in Peds LS	3	LS onset
**C.20.HLAB**	Higher in Adult LS	3	LS onset
**C.20.UQCRB**	Higher in Adult LS	3	LS onset
**C.20.BTF3**	Higher in Adult LS	3	LS onset
**C.31.ENO1**	Higher in Adult LS	6	LS onset
**C.31.CDC42**	Higher in Adult LS	6	LS onset
**C.31.RSRP1**	Higher in Adult LS	6	LS onset
**C.31.SH3BGRL3**	Higher in Adult LS	6	LS onset
**C.31.CD52**	Higher in Adult LS	6	LS onset
**C.31.NDUFS5**	Higher in Adult LS	6	LS onset
**C.31.YBX1**	Higher in Adult LS	6	LS onset
**C.31.PRDX1**	Higher in Adult LS	6	LS onset
**C.31.TMEM59**	Higher in Adult LS	6	LS onset
**C.31.SERBP1**	Higher in Adult LS	6	LS onset
**C.7.PLVAP**	Higher in Adult LS	6	LS onset

Supplemental Table 5: Additional SLIDE latent factors from model used to regress mLoSSI score for LS patients. Additional latent factors chosen by SLIDE for regressing mLoSSI score in combined pediatric and adult LS samples. Latent factor number is used solely to indicate gene membership and does not correspond to any ranking.

Gene	Association	latent factor number	model
**C.21.AQP5**	Lower MRSS	2	LS LoSAI
**C.21.GAPDH**	Lower MRSS	2	LS LoSAI
**C.21.LDHA**	Lower MRSS	2	LS LoSAI
**C.21.KRT15**	Lower MRSS	2	LS LoSAI
**C.21.PDCD4**	Lower MRSS	2	LS LoSAI
**C.21.CLDN10**	Lower MRSS	2	LS LoSAI
**C.21.KRT18**	Lower MRSS	2	LS LoSAI
**C.21.KRT8**	Lower MRSS	2	LS LoSAI
**C.21.DBI**	Lower MRSS	2	LS LoSAI
**C.21.NDRG2**	Lower MRSS	2	LS LoSAI
**C.25.CALML5**	Higher MRSS	2	LS LoSAI
**C.25.ANXA2**	Higher MRSS	2	LS LoSAI
**C.26.ANGPT2**	Higher MRSS	2	LS LoSAI
**C.21.COX7C**	Lower MRSS	3	LS LoSAI
**C.21.NDUFA4**	Lower MRSS	3	LS LoSAI
**C.21.FXYD3**	Lower MRSS	3	LS LoSAI
**C.21.ELOB**	Lower MRSS	3	LS LoSAI
**C.26.ANGPT2**	Higher MRSS	3	LS LoSAI
**C.21.KRT7**	Lower MRSS	3	LS LoSAI
**C.25.CALML5**	Higher MRSS	3	LS LoSAI
**C.25.UBC**	Higher MRSS	5	LS LoSAI
**C.25.GAPDH**	Higher MRSS	5	LS LoSAI
**C.25.NFKBIA**	Higher MRSS	5	LS LoSAI
**C.25.PTMA**	Higher MRSS	5	LS LoSAI
**C.25.UQCRB**	Higher MRSS	5	LS LoSAI
